# Supplementary material for: Supporting the Wound Healing Process—Curcumin, Resveratrol and Baicalin in In Vitro Wound Healing Studies
Source: Pharmaceuticals (Basel). 2023 Jan 6;16(1):82. doi: 10.3390/ph16010082 (PMC9861488; doi:10.3390/ph16010082)
Supplement: Supplementary file 1 [file pharmaceuticals-16-00082-s001.zip › pharmaceuticals-2109651-supplementary.pdf]

Table S1. MTT results for curcumin – Balb3t3

Plate number	Concentration of curcumin [µg/mL]									
	0	0	0.5	1	3	6	9	12	20	25
	Absorbance									
515	0.663	0.873	0.747	0.759	0.779	0.686	0.674	0.654	0.736	0.678
	0.656	0.908	0.793	0.846	0.726	0.763	0.703	0.726	0.658	0.592
	0.683	0.884	0.738	0.831	0.766	0.710	0.717	0.662	0.654	0.478
	0.652	0.874	0.823	0.798	0.706	0.641	0.649	0.702	0.644	0.544
	0.689	0.891	0.739	0.748	0.814	0.726	0.669	0.564	0.547	0.490
	0.860	0.781	0.677	0.776	0.847	0.833	0.709	0.669	0.677	0.383
517	0.783	0.775	0.803	0.785	0.631	0.751	0.797	0.763	0.725	0.626
	0.563	0.733	0.865	0.878	0.790	0.560	0.854	0.822	0.782	0.727
	0.682	0.721	0.936	0.764	0.643	0.594	0.694	0.779	0.708	0.627
	0.681	0.750	0.869	0.748	0.847	0.712	0.744	0.749	0.629	0.566
	0.726	0.875	0.686	0.770	0.765	0.659	0.718	0.759	0.767	0.528
	0.912	0.763	0.794	0.767	0.669	0.799	0.878	0.977	0.883	0.638
516	0.726	0.789	0.997	0.950	0.970	0.959	0.879	0.733	0.774	0.981
	0.808	0.890	0.952	0.863	0.927	0.971	0.912	1.015	1.105	0.973
	0.745	0.739	0.842	0.912	0.975	0.766	0.590	0.631	0.661	0.965
	0.988	1.012	1.045	0.951	1.011	0.944	0.881	0.891	0.927	0.996
	0.897	0.946	0.964	1.007	0.785	1.040	0.854	0.918	1.042	1.001
	0.950	0.913	1.047	0.992	0.988	0.930	0.911	0.849	0.934	0.667

Table S2. MTT results for resveratrol – Balb3t3

Plate number	Concentration of resveratrol [µg/mL]									
	0	0	0.5	1	3	6	9	12	20	25
	Absorbance									
489	0.468	0.389	0.363	0.406	0.485	0.412	0.324	0.460	0.279	0.394
	0.302	0.443	0.392	0.392	0.499	0.442	0.427	0.398	0.440	0.368
	0.365	0.382	0.371	0.450	0.564	0.336	0.434	0.512	0.392	0.282
	0.352	0.362	0.338	0.398	0.479	0.386	0.444	0.518	0.293	0.298
	0.445	0.490	0.415	0.390	0.433	0.484	0.400	0.483	0.466	0.288
	0.495	0.500	0.489	0.522	0.385	0.473	0.378	0.317	0.372	0.316
485	0.220	0.446	0.514	0.513	0.393	0.481	0.499	0.405	0.446	0.366
	0.318	0.387	0.370	0.420	0.445	0.459	0.403	0.374	0.421	0.386
	0.659	0.545	0.417	0.439	0.400	0.389	0.519	0.420	0.431	0.384
	0.387	0.656	0.430	0.490	0.369	0.456	0.541	0.456	0.427	0.305
	0.533	0.612	0.468	0.467	0.542	0.463	0.479	0.402	0.325	0.306
	0.517	0.604	0.379	0.426	0.485	0.522	0.592	0.391	0.334	0.271
488	0.353	0.424	0.504	0.396	0.338	0.403	0.365	0.287	0.318	0.249
	0.339	0.393	0.469	0.437	0.403	0.311	0.370	0.316	0.264	0.286
	0.421	0.491	0.444	0.442	0.343	0.375	0.289	0.334	0.305	0.352
	0.325	0.325	0.428	0.388	0.476	0.462	0.289	0.290	0.299	0.340
	0.396	0.430	0.464	0.316	0.363	0.345	0.378	0.280	0.287	0.261
	0.408	0.436	0.425	0.449	0.341	0.452	0.318	0.335	0.259	0.270

Table S3. MTT results for baicalin – Balb3t3

Plate number	Concentration of baicalin [µg/mL]									
	0	0	0.5	1	3	6	9	12	20	25
	Absorbance									
490	0.135	0.173	0.162	0.121	0.110	Er	0.099	0.108	0.113	0.103
	0.182	0.141	0.170	0.218	0.117	0.101	0.097	0.102	0.101	0.103
	Er	0.154	0.142	0.183	0.109	0.120	0.108	0.095	0.097	0.117
	0.121	0.152	0.145	0.140	0.127	0.104	0.111	0.122	0.114	0.112
	0.168	0.138	0.169	0.159	0.133	0.161	0.107	0.105	0.123	0.117
	0.168	0.153	Er	0.167	0.139	0.145	0.162	0.153	0.105	0.121
495	0.155	0.136	0.157	0.158	0.127	0.111	0.123	0.105	0.117	0.090
	0.159	0.161	0.146	0.122	0.121	0.108	0.133	0.159	0.155	0.095
	0.160	0.168	0.159	0.160	0.129	0.149	0.189	0.166	0.156	0.123
	0.185	0.135	0.128	0.209	0.153	0.153	0.170	0.137	0.121	0.139
	0.149	0.154	0.161	0.237	0.160	0.193	0.229	0.180	0.161	0.175
	0.155	0.182	0.115	0.158	0.147	0.171	0.199	0.145	0.182	0.169
494	0.190	0.147	0.178	0.214	0.179	0.144	0.127	0.119	0.101	0.119
	0.160	0.150	0.185	0.192	0.170	0.194	0.159	0.120	0.135	0.126
	0.172	0.178	0.174	0.200	0.176	0.189	0.176	0.168	0.212	0.139
	0.159	0.121	0.183	0.200	0.164	0.159	0.173	0.202	0.145	0.168
	0.134	0.124	0.180	0.211	0.205	0.181	0.160	0.179	0.202	0.170
	0.199	0.095	0.176	0.197	0.170	0.212	0.251	0.145	0.172	0.117

Table S4. MTT results for curcumin – L929

Plate number	Concentration of curcumin [µg/mL]									
	0	0	0.5	1	3	6	9	12	20	25
	Absorbance									
518	0.211	0.237	0.235	0.177	0.235	0.195	0.202	0.152	0.175	0.130
	0.180	0.227	0.205	0.234	0.229	0.205	0.194	0.164	0.153	0.103
	0.228	0.212	0.204	0.228	0.216	0.262	0.186	0.162	0.150	0.093
	0.220	0.219	0.244	0.228	0.286	0.186	0.269	0.447	0.179	0.142
	0.231	0.271	0.244	0.244	0.189	0.204	0.265	0.204	0.194	0.164
	0.228	0.210	0.228	0.227	0.241	0.480	0.392	0.246	0.206	0.236
519	Er	Er	0.166	0.159	0.195	0.192	0.139	0.195	0.156	0.136
	0.160	0.148	0.179	0.206	0.261	0.297	0.328	0.142	0.139	0.118
	0.175	0.166	0.285	0.192	0.395	0.208	0.166	0.167	0.145	0.132
	0.188	0.201	0.231	0.208	0.228	0.267	0.156	0.131	0.131	0.106
	0.195	0.173	0.220	0.199	0.362	0.223	0.168	0.181	0.152	0.114
	0.237	0.197	0.209	0.228	0.638	0.391	0.193	0.165	0.168	0.160

Table S5. MTT results for resveratrol– L929

Plate number	Concentration of resveratrol [µg/mL]									
	0	0	0.5	1	3	6	9	12	20	25
	Absorbance									
489	0.444	Er	0.485	0.503	0.522	0.450	0.525	0.460	0.368	0.398
	0.445	0.453	0.445	0.451	0.469	0.492	0.494	0.459	0.613	0.494

	0.579	0.571	0.452	0.432	0.510	0.684	0.547	0.508	0.503	0.452
	0.487	0.391	0.401	0.448	0.420	0.554	0.634	0.531	0.387	0.278
	0.505	0.518	0.404	0.357	0.375	0.444	0.542	0.580	0.320	0.396
	0.545	0.323	0.405	0.414	0.306	0.424	0.580	0.638	0.332	0.285
485	0.396	0.507	0.692	0.586	0.574	0.586	0.422	0.523	0.507	0.407
	0.462	0.553	0.538	0.632	0.621	0.551	0.487	0.550	0.363	0.434
	0.541	0.627	0.589	0.548	0.701	0.428	0.511	0.421	0.412	0.416
	0.462	0.498	0.621	0.606	0.592	0.481	0.456	0.432	0.463	0.392
	0.478	0.526	0.581	0.511	0.488	0.505	0.539	0.498	0.368	0.349
	0.431	0.482	0.620	0.567	0.425	0.474	0.473	0.364	0.441	0.306
488	0.379	Er	0.440	0.308	0.362	0.313	0.365	0.337	0.414	Er
	0.388	Er	0.454	0.380	0.392	0.411	0.353	0.348	0.342	Er
	0.501	Er	0.409	0.603	0.422	0.394	0.309	0.424	0.356	Er
	0.379	Er	0.360	0.388	0.411	0.401	0.404	0.338	0.503	Er
	0.592	Er	0.261	0.413	0.377	0.473	0.419	0.285	0.393	Er
	0.454	Er	0.418	0.419	0.451	0.374	0.286	0.359	0.403	Er

Table S6. MTT results for baicalin – L929

Plate number	Concentration of baicalin [μg/mL]									
	0	0	0.5	1	3	6	9	12	20	25
	Absorbance									
493	0.120	0.110	Er	0.126	0.099	0.091	0.101	0.133	0.101	0.101
	Er	0.143	0.133	0.109	0.122	0.099	0.096	0.144	0.165	0.093
	0.104	0.110	0.152	0.153	0.093	0.089	0.099	0.095	0.104	0.100

	0.139	0.109	0.135	0.156	0.127	0.107	0.131	0.140	0.114	0.118
	0.124	0.110	0.151	0.143	0.163	0.125	Er	0.113	0.124	0.104
	0.138	0.118	0.177	0.135	0.183	0.114	0.130	0.109	0.113	0.102
492	0.118	0.101	0.116	0.140	0.097	0.088	0.093	0.092	0.089	0.085
	0.131	0.101	0.125	0.115	0.105	0.106	0.120	0.094	0.109	0.072
	0.107	0.141	0.125	0.100	0.102	0.080	0.100	0.080	0.081	0.077
	0.101	0.101	0.139	0.114	0.129	0.095	0.120	0.095	0.090	0.082
	0.114	0.110	0.174	0.149	0.125	0.108	0.112	Er	0.093	0.084
	0.116	0.095	0.143	0.107	0.107	0.106	0.095	0.102	0.102	0.085
491	0.117	0.125	0.131	0.122	0.100	0.089	0.099	0.088	0.083	0.095
	0.112	0.138	0.129	0.124	0.124	0.098	0.127	0.094	0.087	0.082
	0.103	0.110	0.135	0.088	0.108	0.104	0.112	0.088	0.083	0.081
	0.107	0.137	0.129	0.119	0.109	0.086	0.122	0.095	0.090	0.079
	0.131	0.133	0.130	0.113	0.100	0.111	0.109	0.096	0.125	0.083
	0.119	0.149	0.154	0.148	0.106	0.082	Er	0.105	0.132	0.110

Er - error disqualifying the sample result

Table S7. ST results for curcumin – Balb3t3

Concentration [µg/mL]	0		0.5		1		2		4		6		9		12		20	
Time [h]	0	24	0	24	0	24	0	24	0	24	0	24	0	24	0	24	0	24
Measure 1 [µm]	68	63	75	58	73	60	75	40	71	34	78	43	72	39	76	60	80	70
Measure 2 [µm]	74	61	71	51	70	59	71	40	53	32	77	42	74	42	71	52	71	65
Measure 3 [µm]	65	59	77	60	72	63	70	33	65	29	75	45	76	41	73	54	72	69

Table S8. ST results for resveratrol – Balb3t3

Concentration [µg/mL]	0	0.5	1	2	4	6	9	12	20

Time [h]	0	24	0	24	0	24	0	24	0	24	0	24	0	24	0	24	0	24
Measure 1 [µm]	93	58	99	22	85	15	69	9	77	15	80	59	72	51	71	64	75	65
Measure 2 [µm]	79	70	93	14	70	14	81	13	79	19	90	64	71	50	73	69	70	63
Measure 3 [µm]	88	67	90	16	85	22	75	5	84	21	93	84	75	59	70	65	80	79

Table S9. ST results for baicalin – Balb3t3

Concentration [µg/mL]	0		0.5		1		2		4		6		9		12		20	
Time [h]	0	24	0	24	0	24	0	24	0	24	0	24	0	24	0	24	0	24
Measure 1 [µm]	70	60	77	36	83	25	81	28	69	14	70	22	72	37	70	41	76	44
Measure 2 [µm]	73	59	78	32	75	30	75	16	73	9	77	22	77	42	73	41	73	45
Measure 3 [µm]	71	65	71	37	71	25	70	12	70	11	74	10	73	30	80	38	78	46

Table S10. ST results for curcumin – L929

Concentration [µg/mL]	0		0.5		1		2		4		6		9		12		20	
Time [h]	0	24	0	24	0	24	0	24	0	24	0	24	0	24	0	24	0	24
Measure 1 [µm]	70	60	73	59	71	61	69	39	70	37	71	38	67	40	70	57	74	69
Measure 2 [µm]	72	55	69	57	70	58	68	44	71	40	69	45	69	38	74	54	71	67
Measure 3 [µm]	67	63	71	59	72	60	73	37	68	35	73	48	70	35	73	58	74	70

Table S11. ST results for resveratrol – L929

Concentration [µg/mL]	0		0.5		1		2		4		6		9		12		20	
Time [h]	0	24	0	24	0	24	0	24	0	24	0	24	0	24	0	24	0	24
Measure 1 [µm]	90	59	92	21	88	17	70	11	75	13	83	63	69	47	70	62	72	68
Measure 2 [µm]	77	69	81	12	78	12	79	9	77	17	92	60	71	47	70	68	90	72
Measure 3 [µm]	78	70	77	18	81	15	83	5	82	20	74	70	70	55	73	68	85	77

Table S12. ST results for baicalin – L929

Concentration [µg/mL]	0		0.5		1		2		4		6		9		12		20	
Time [h]	0	24	0	24	0	24	0	24	0	24	0	24	0	24	0	24	0	24
Measure 1 [µm]	73	61	72	31	81	29	71	23	69	15	73	20	79	35	80	41	80	41
Measure 2 [µm]	72	57	81	29	79	37	73	17	78	7	70	17	81	40	80	38	71	40
Measure 3 [µm]	80	69	78	35	70	28	70	17	80	12	71	10	85	35	83	39	70	42

Table S13. Anderson – Darling test results

cell line	Substance	Concentration [µg/mL]	*p* - value	The hypothesis of normality is: 0 = NOT rejected; 1 = rejected for significance level α		
				α = 0.05	α = 0.025	α = 0.01
Balb3t3	Curcumin	control	0.223	0	0	0
		0.5	0.660	0	0	0
		1	0.553	0	0	0
	Resveratrol	control	0.608	0	0	0
		0.5	0.607	0	0	0
		1	0.800	0	0	0
	Baicalin	control	0.869	0	0	0
		0.5	0.376	0	0	0
		1	0.238	0	0	0
L929	Curcumin	control	0.236	0	0	0
		3	0.002	1	1	1
		6	0.040	1	0	0
	Resveratrol	control	0.968	0	0	0
		3	0.750	0	0	0
		6	0.167	0	0	0
	Baicalin	control	0.034	1	0	0
		0.5	0.009	1	1	1
		1	0.424	0	0	0

Table S14. T-test results for all bioflavonoids

Concentration of bioflavonoid [µg/mL]	0.5	1	3	6	9	12	20	25
Balb/3t3								

Curcumin	0.0733	0.1025	0.670	0.4211	0.2873	0.363	0.3126	0.0025
Resveratrol	0.8901	0.9214	0.9528	0.7263	0.3087	Not tested	0.0007	2.28e-6
Baicalin	0.3558	0.0016	0.2556	0.8051	0.9349	0.0572	0.1321	Not tested
L929								
Curcumin	0.0762	0.3382	Not tested	0.0085	Not tested	Not tested	6.53e-6	1.58e-7
Resveratrol	0.9702	0.9869	0.7076	0.7486	0.7043	0.8166	Not tested	0.0039
Baicalin	Not tested	0.1173	Not tested	6.81e-6	Not tested	Not tested	0.0051	1.09e-9
